# Rising incidence of *Enterococcus* species in microbiological specimens from orthopedic patients correlates to increased use of cefuroxime: A study concentrating on tissue samples

**DOI:** 10.3109/17453674.2013.792028

**Published:** 2013-05-31

**Authors:** Peter Christian Siesing, Jens Peter Alva-Jørgensen, Jakob Brodersen, Magnus Arpi, Poul Einar Jensen

**Affiliations:** ^1^Departments of Orthopedic Surgery; ^2^Clinical Microbiology, Copenhagen University, Herlev Hospital, Denmark; ^3^Department of Fish Ecology and Evolution, Centre of Ecology, Evolution and Biochemistry, EAWAG Swiss Federal Institute of Aquatic Science and Technology, Kastanienbaum, Switzerland.

## Abstract

**Background and purpose:**

Enterococci are emerging causes of severe infections, including wound and bone infections in orthopedic patients. The main purpose of this study was to determine whether there was a correlation between the incidence of enterococci in tissue samples (biopsies) from orthopedic patients and consumption of cefuroxime in the orthopedic department.

**Methods and results:**

Data were obtained from the department of clinical microbiology and the hospital pharmacy. The consumption of cefuroxime successively increased from 40 defined daily doses (DDD)/10^3^ bed days in 2002 to 212 DDD/10^3^ bed days in 2009. The incidence of patients with enterococci in tissue samples increased steadily from 1.03/10^3^ bed days in 2002 to 5.90/10^3^ bed days in 2009. Regression analysis revealed a significant correlation between the consumption of cefuroxime and the incidence of enterococci.

**Interpretation:**

Continuous surveillance of species distribution, resistance rates, and antibiotic consumption is of utmost importance for optimal antibiotic strategy in orthopedic patients.

There is a well-documented global increase in multiresistant microorganisms as causes of different clinical infections. This increase is often correlated to a high consumption of broad-spectrum antibiotics, especially cephalosporins and fluoroquinolones (ESAC 2010). Furthermore, enterococci are now reported to be the third leading cause of nosocomial infections (Hidron et al. 2008). The same tendency has also been observed in Denmark, where the total consumption of cephalosporins and fluoroquinolones has increased 3-fold in the 10-year period 2000–2009 (DANMAP 2009).

The reason for the emerging enterococcal infections is not fully understood, but an important contributory factor is probably the selection pressure from increasing consumption of cephalosporins. This promotes enterococci, which are all inherently resistant to this group of antibiotics (Pallares et al. 1993, Leclerq and Courvalin 2005). For years, penicillinase-stable penicillins and first-generation cephalosporins have been the preferred antibiotics for treatment and prophylaxis in orthopedic patients. Today, cefuroxime (a second-generation cephalosporin) is widely and increasingly used in orthopedic departments in many countries (Engesæter et al. 2003). The Danish Orthopaedic Society recommends cefuroxime prophylaxis for prosthetic surgery and osteosynthesis (DOS 2010).

In this retrospective study, we investigated whether there was a correlation between the rising incidence of enterococcal infections and antibiotic consumption in the orthopedic department at Herlev University Hospital. Our findings could have consequences for the future choice of strategy for antibiotic treatment and prophylaxis in orthopedic patients.

## Materials and methods

Microbiological data were obtained from the laboratory database at the Department of Clinical Microbiology, Herlev University Hospital. The following data were retrieved for patients admitted to the orthopedic department: identity of patient, type of specimen (including total number of tissue cultures), sampling date, culture results, and results of susceptibility testing.

Tissue culture results from the orthopedic department in the 20-year period 1990–2009 were divided into 5 periods of 4 years to show changes in species distribution over time. Differentiation between bone and soft tissue biopsies was not possible in this study. All the cultures from tissue where included irrespective of the diagnosis of the patient. The 12 most frequent bacterial species or bacterial groups were selected for analysis of changes in species distribution over time. For every patient, only 1 incident was included. An incident was defined as isolation of a microorganism in 1 or more samples from the same operation. If patients underwent more than 1 operation within 6 months and the microbiology was the same, it was included as 1 incident.

Antibiotic consumption in the orthopedic department was available for each year during the period 2002–2010 (data provided by the hospital pharmacy). Antibiotic consumption was reported as defined daily doses (DDD) (ATC/DDD 2011) per 10^3^ occupied bed days. Consumption of cefuroxime is reported, as this was the only cephalosporin used in the department.

The incidence of *Enterococcus* spp. was calculated as the number of patients with *Enterococcus* species in tissue samples per 10^3^ occupied bed days for the period 2002–2010. Only 1 episode per patient was recorded.

Possible correlation between cefuroxime and other antibiotics on the one hand and incidence of *Enterococcus* on the other was only analyzed for the period during which data for antibiotic consumption were available (2002–2010).

### Statistics

Statistical analysis was performed with chi-square test, linear regression analysis, and multiple regression analysis. For linear and multiple linear regression, we tested for deviation of distributions of residuals from a normal distribution using the Shapiro-Wilk test. In no case was the distribution significantly different from a normal distribution (p = 0.5 and p = 0.2, respectively).

For analysis of the relationship between the incidence of *Enterococcus* in tissue samples from the orthopedic department (dependent variable) and the consumption of 4 different types of antibiotics (independent variables), we used multiple linear regression analysis. Also, time (year) was included in this analysis as an independent variable, to control for any temporal trends unrelated to antibiotic treatment. Multiple linear regression analysis was performed using backward selection with p < 0.1 as a selection criteria. Statistical tests were performed using SPSS software version 17.0.

## Results

The numbers of patients with enterococci in tissue samples and the relative proportion of all isolates that were enterococci were markedly higher in the last 2 four-year periods (Table 1). The incidence of patients with enterococci in tissue samples increased steadily from 1.03 in 2002 to 5.9 in 2009. A slight fall to 5.6 was observed in 2010 (Table 2). The vast majority (99%) of enterococci were *E. faecalis* and *E. faecium.* Comparing these 2 species, the proportion of *E. faecium* increased from 7% in the first 3-year period to 15% in the last 3-year period (p < 0.001).

**Table 1.** Time trends in species distribution in partients with positive tissue cultures

Period	1990–1993		1994–1997		1998–2001		2002–2005		2006–2009	
Microorganism	No. of patients	Percent	No. of patients	Percent	No. of patients	Percent	No. of patients	Percent	No. of patients	Percent
*Stapylococcus aureus*	27	47	38	46	35	43	74	53.5	136	51.5
Coagulase-negative staphylococcus	15	26	26	32	28	34	51	37	78	29.5
Beta-hemolytic streptococcus	4	7	8	10	6	7.5	8	6	21	8
Non-hemolytic streptococcus	2	3.5	2	2.5	1	1	2	1.5	4	1.5
*Enterococcus* species	11	19	10	12	10	12	33	24	58	22
*Corynebacterium* species	5	9	9	11	13	16	9	6.5	23	85
*E. coli*	2	3.5	7	8.5	10	12	7	5	24	9
*Enterobacter* species	6	10.5	1	1	0	0	0	0	20	7.5
Other Enterobactericeae	10	17.5	14	17	9	11	41	30	48	18
Anaerobic bacteria	3	5	7	8.5	6	7.5	21	15	36	13.5
*Candida* species	0	0	1	1	1	1	0	0	2	1
Other types	8	14	7	8.5	8	10	16	11.5	45	17
Total	93		129		127		270		495	

**Table 2.** Number of patients and incidence of isolation of enterococci from clinical specimens

Year	2002	2003	2004	2005	2006	2007	2008	2009	2010
Orthopedic department									
No. of patients with enterococci in tissue samples	18	14	19	32	22	48	85	136	119
Incidence **^a^** of patients with enterococci in tissue samples	1.03	0.8	1.2	2.1	1.5	3.5	4.5	5.9	5.6
Total hospital									
No. of patients with enterococci (all specimens)	906	1,053	1,245	1,420	1,506	1,690	1,973	2,273	2,020
Incidence **^a^** of patients with enterococci (all specimens)	4.5	5.4	6.3	7.3	7.9	9.1	9.2	10.0	9.9

**^a^** No. of patients/10^3^ bed days.

The consumption of cefuroxime increased from 40 DDD/10^3^ bed days in 2002 to 212 DDD/10^3^ bed days in 2009 (Figure). In 2009, the consumption of cefuroxime exceeded the consumption of dicloxacillin in the orthopedic department.

**Figure:**
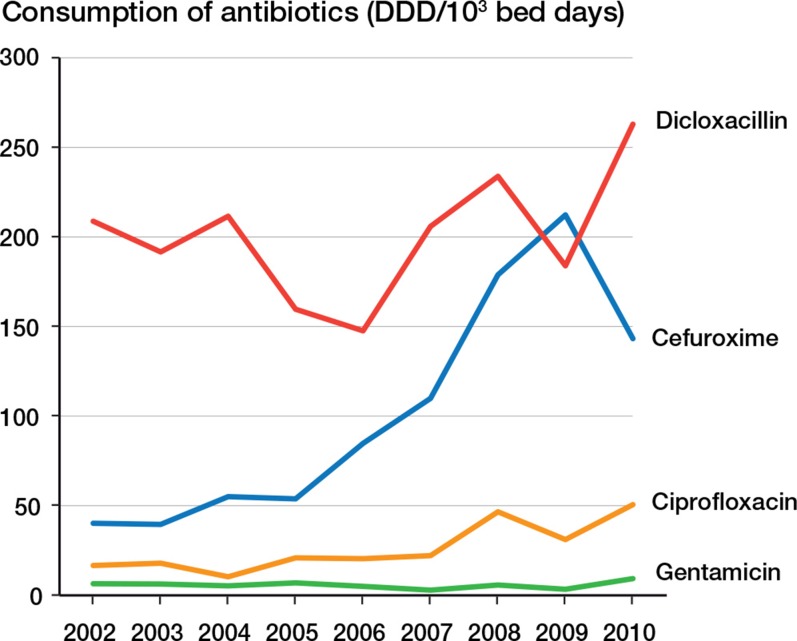
Consumption of antibiotics as defined daily doses (DDD) at the Department of Orthopedic Surgery, Herlev University Hospital, 2002–2010.

Linear regression analysis revealed a significant relationship between the consumption of cefuroxime and the incidence of *Enterococcus* in tissue samples (B = 0.68 (95% CI: 0.43–0.93); p < 0.001).

Multiple regression analysis revealed that of the variables included in the test, consumption of cefuroxime was the only variable statistically significantly correlated to the increasing incidence of enterococci (B = 0.73 (95% CI: 0.52–0.94); p < 0.001). There were no statistically significant correlations between dicloxacillin (95% CI (B): –0.53 to 0.84; p = 0.5), gentamicin (95% CI (B): –0.39 to 14; p = 0.06), and ciprofloxacin (95% CI (B): –4.6 to 1.6; p = 0.3) consumption on the one hand and an increasing incidence of enterococci on the other.

## Discussion

From 1990 to 2009, there was an increasing incidence of enterococci in tissue biopsies, and from 2002 to 2010 there was a dramatic increase. The reason for this is unclear, but an important contributory factor could be the selection pressure of increasing consumption of cephalosporins. This promotes enterococci, which are inherently resistant to this group of antibiotics (Pallares et al. 1993, Leclerq and Courvalin 2005).

The increased use of cefuroxime in the orthopedic department is probably caused by several factors. The encatchment area of the hospital has increased, leading to a larger patient population. Considerably more patients with trauma, patients needing amputation, and patients with deep infections are admitted. Though we corrected for the number of bed days, cefuroxime consumption increased more than 3-fold in the period 2002–2010 (Figure). In 2010, the antibiotic prophylaxis given prior to surgery was changed, from cefuroxime to dicloxacillin and gentamicin. At the moment, we are waiting to see if a reduction in the incidence of *Enterococcus* occurs.

We found a correlation between the increasing use of cefuroxime and the increased incidence of *Enterococcus* species in tissue samples. Multiple regression analysis showed that cefuroxime was the only one of the antibiotics tested that had a statistically significant effect on the increase in enterococci, which was not the case for dicloxacillin, gentamicin, and ciprofloxacin. The correlation between increased consumption of cephalosporins and increased incidence of enterococci applied both at the hospital level and at the departmental level, highlighting the selection pressure of high consumption of cephalosporins.

The rising incidence of enterococci in biopsies from orthopedic patients is worrying, because of the limited number of antibiotics available with sufficient activity against this species. Enterococci are resistant to cephalosporins, penicillinase-resistant penicillins, clinically achievable concentrations of gentamicin, clindamycin, and trimethoprim-sulfamethoxazole, and they are relatively resistant to fluoroquinolones (Leclerq and Courvalin 2005). *E. faecalis* is susceptible to ampicillin, whereas the majority of *E. faecium* strains are ampicillin resistant.

Although most enterococci are susceptible to rifampicin, this agent is bacteriostatic against most strains of enterococci (Morris et al. 1993) and must be given in combination with another antibiotic to avoid development of resistance (Van der Auwera et al. 1983, Kadudugamuwa et al. 2004). Vancomycin generally has good in vitro activity against enterococci, but a rising incidence of vancomycin-resistant enterococci has been reported in several studies (Rice 2001, EARSS 2009). Vancomycin has relatively poor penetration of bone tissue (Graziani et al. 1988), and considering the minimal inhibitory concentrations of vancomycin against enterococci, the pharmacokinetic and pharmacodynamic profile of vancomycin for treatment of enterococcal bone infections is not favorable. Selection of enterococci in an orthopedic department is therefore very unfortunate because of the few therapeutic possibilities for treating deep orthopedic enterococcal infections, especially in patients with penicillin allergy.

We found an increasing proportion of *E. faecium* in enterococcal isolates, from 7% to 15%. This tendency has been reported by others (DANMAP 2009). *E. faecium* is resistant to all β-lactam antibiotics, and vancomycin and linezolide are often the only effective antibiotics available. It is well documented that a high consumption of carbapenems, e.g. meropenem, selects for *E. faecium* (Zhanel et al. 2007, Meyer et al. 2010). Allthough the carbapenem consumption at the orthopedic department was low, consumption of carbapenems in the entire hospital increased 4-fold in the period 2002–2010. This may have contributed to the increasing proportion of *E. faecium *isolates in the orthopedic department.

In summary, the high and increasing consumption of cefuroxime over several years in the orthopedic department at our hospital was correlated to a rising incidence of patients with enterococci in tissue samples. Selection of enterococci by treatment with cefuroxime is problematic, because they are resistant to penicillinase-resistant penicillins, clindamycin, and achievable levels of aminoglycosides. Rifampicin only has a bacteriostatic effect against most enterococci. These agents are important in the armamentarium for treatment of deep-seated orthopedic infections. A continuous local surveillance of species distribution, resistance rates, and antibiotic consumption is of prime importance for an optimal antibiotic strategy in orthopedic patients.
